# Challenges in PT/INR harmonization across the total testing process

**DOI:** 10.11613/BM.2026.030504

**Published:** 2026-10-15

**Authors:** Jia-min Zou, Tian-ji Li, Qi Liu, Yong-gang Hu

**Affiliations:** 1 Department of Clinical Laboratory, Luzhou Mental Hospital, Luzhou, China; 2 Department of Orthopedics, People’s Hospital of Naxi District, Luzhou, China; 3 Department of Clinical Laboratory, People’s Hospital of Naxi District, Luzhou, China

**Keywords:** prothrombin time, international normalized ratio, harmonization, standardization, direct oral anticoagulants

## Abstract

The International Normalized Ratio (INR), introduced by the World Health Organization in 1983, is central to vitamin K antagonist (VKA) therapy monitoring. However, thromboplastin standardization alone does not ensure harmonized prothrombin time (PT)/INR results. Residual variability arises throughout the testing continuum. In this narrative review, we examine the principal sources of residual PT/INR non-equivalence across the “vein-to-report” pathway. Particular attention is devoted to clinical settings in which the assumptions underlying INR calibration are only partially satisfied, including advanced liver disease and exposure to direct oral anticoagulants. We further discuss postanalytical factors, such as determination of the mean normal prothrombin time and system-specific verification of the International Sensitivity Index, as well as quality assurance strategies, including the use of commutable external quality assessment materials and patient-based real-time quality control. Prothrombin time/INR harmonization requires integrating preanalytical practices, recognizing reagent-instrument dependencies, and quality strategies reflecting real-world testing conditions.

## Graphical abstract

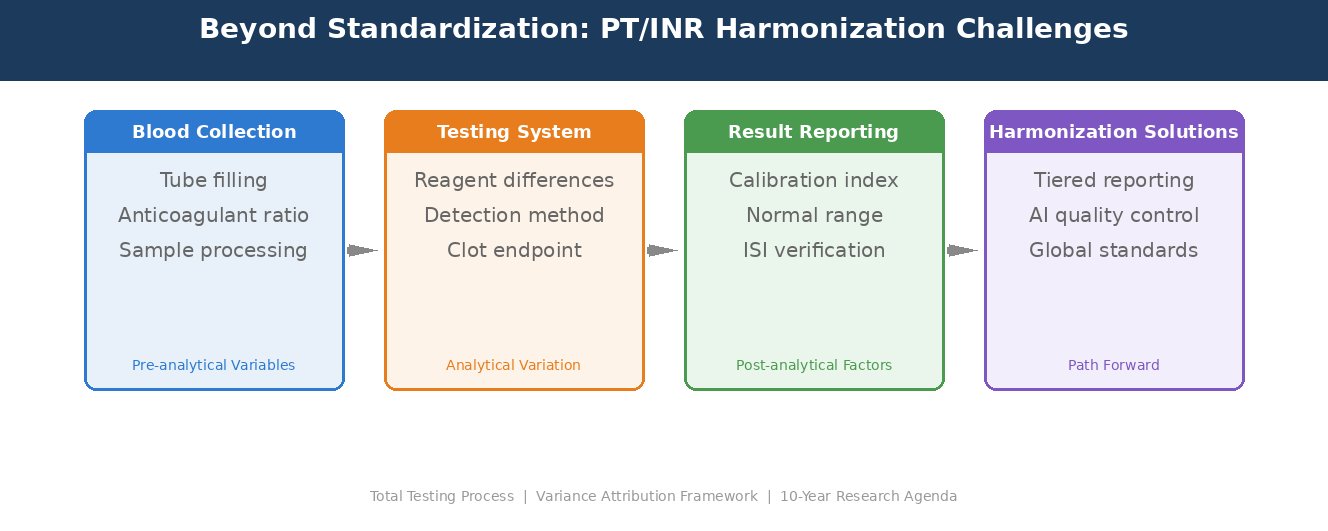

## Highlights

• Prothrombin time/international normalized ratio (PT/INR) non-equivalence persists despite thromboplastin calibration due to variability across the total testing process

• INR does not reliably harmonize results in liver disease or direct oral anticoagulant exposure

• Reagent–instrument combinations introduce system-dependent biases not fully corrected by standard calibration

• Commutable external quality assessment materials and patient-based real-time quality control are essential for detecting clinically relevant bias

• True PT/INR harmonization requires management of the entire vein-to-report pathway

## Introduction

The most well-established preanalytical determinants of prothrombin time/international normalized ratio (PT/INR) reliability originate from specimen collection. Proper venipuncture technique is essential to avoid local activation or contamination. Prolonged tourniquet application, traumatic venipuncture, incorrect collection order, and sampling through indwelling lines may compromise sample quality and introduce variability in clot-based assays (*1*, *2*).

The International Normalized Ratio, introduced by the World Health Organization (WHO) in 1983, uses the International Sensitivity Index (ISI) to reduce thromboplastin-related differences and establish metrological traceability (*3*, *4*). Despite being one of the most influential standardization tools in laboratory medicine, “standardization” has not fully translated into “harmonization” - clinically equivalent results across all methods and settings (*5*-*8*).

Analytical variability also stems from how clot formation is detected. Prothrombin time systems rely on mechanical or viscosity detection, or optical monitoring of light transmission changes during fibrin formation. These methods differ not only in physical basis but also in sensitivity to hemolysis, lipemia, icterus, hypofibrinogenemia, and other matrix abnormalities (*9*, *10*). Even when the same specimen is analyzed with closely related reagent systems, inconsistencies may persist because the clotting endpoint is operationally assigned rather than universally observed. In optical systems, the reported PT may depend on signal thresholds, clotting-curve processing rules, and platform-specific endpoint algorithms (*11*-*13*). Such differences may have clinical consequences when PT/INR results are used for vitamin K antagonist dose adjustment, interpretation of direct oral anticoagulant exposure, liver-disease severity assessment, or comparison of results across institutions.

The aim of this narrative review is therefore to critically examine residual PT/INR non-equivalence across the total testing process. We focus on how preanalytical conditions, reagent-instrument interactions, endpoint-detection principles, postanalytical INR parameters, external quality assessment materials, and clinical context influence the degree to which PT/INR results can be considered clinically interchangeable. By linking these sources of variability to patient-management consequences, we seek to clarify why PT/INR harmonization requires more than correct application of the INR formula and why system-specific verification and context-aware interpretation remain necessary.

### Prothrombin time and International Normalized Ratio

The expanding use of direct oral anticoagulants (DOACs) illustrates an important boundary of PT/INR harmonization beyond the vitamin K antagonist (VKA) model. Unlike VKAs, which reduce multiple vitamin K-dependent factors in a relatively coordinated manner, DOACs produce target-specific effects that reflect drug class, plasma concentration, and reagent-dependent analytical responsiveness rather than biologically standardized patterns (*14*-*16*). Accordingly, PT/INR results in DOAC-exposed samples should not be interpreted as standardized measures of anticoagulant intensity. The clinical magnitude of this divergence is substantial. Tripodi and colleagues demonstrated that plasma from patients with advanced liver disease produced INR differences of 15-20% when tested with reagents at opposite ends of the ISI spectrum (ISI 0.9 *vs.* ISI 1.7), despite equivalent calibration traceability (*17*).

International Normalized Ratio was introduced to reduce PT variability by transforming it into a standardized expression: INR = (PT_patient_ / MNPT)^ISI^, where MNPT denotes the mean normal prothrombin time and ISI denotes the International Sensitivity Index (*3*, *4*). This transformation improves comparability mainly in patients receiving VKAs, in whom vitamin K-dependent coagulation factors decrease in a relatively coordinated manner. However, INR standardization remains conditional. When this biological assumption is not met, clinically relevant non-equivalence may persist (*18*). To this end, we examine the harmonization of PT/INR from a preanalytical, analytical, and postanalytical perspective, which is primarily structured around a five-tier framework: patient-related biological context and non-parallel factor abnormalities; procedural preanalytical variation; reagent-instrument and endpoint-detection dependence; postanalytical INR parameters, including MNPT determination and ISI verification; and external quality assessment (EQA) commutability and reporting practice.

## Preanalytical phase

The preanalytical phase is a major source of coagulation testing variability. Preanalytical errors and unsuitable samples can account for up to 70-77% of all laboratory errors. In coagulation testing specifically, specimen rejection rates are significantly higher than for other test groups (*19*, *20*). This review distinguishes procedural preanalytical variables (directly affecting specimen quality) from patient-related biological conditions (altering the clinical context of PT/INR interpretation). Only procedural variables are cross-institutional standardization targets; biological modifiers define the limits of interpretive comparability (*21*-*23*).

### Patient-related biological determinants beyond procedural preanalytics

Not all influences preceding PT measurement are procedural. Before venipuncture, the patient’s biological state may already affect both the generation and interpretation of PT prolongation. Unlike classical preanalytical variables, these factors do not primarily impair specimen integrity, but alter the biological context in which PT/INR is produced and interpreted.

This distinction is important because the INR model was developed in VKA-treated patients (*24*). In other conditions, PT prolongation reflects heterogeneous and often overlapping pathophysiological mechanisms. In chronic liver disease, including cirrhosis, alterations involve not only reduced synthesis of procoagulant factors but also concomitant changes in anticoagulant and fibrinolytic pathways; therefore, PT/INR captures only a limited component of the rebalanced hemostatic system. Moreover, because the INR was standardized primarily for monitoring vitamin K antagonist therapy, it does not reliably harmonize PT results across thromboplastin reagents in patients with chronic liver disease. Inter-reagent differences can reach approximately 0.7 INR units in this setting, underscoring that INR should not be used as a stand-alone measure of global coagulopathy or bleeding risk in cirrhosis (*25*). In sepsis, by contrast, PT prolongation is more closely related to inflammation-driven coagulation activation, factor consumption, endothelial dysfunction and consumptive coagulopathy, while reduced vitamin K availability in critical illness may provide a partially overlapping and potentially modifiable contribution (*26*, *27*).

From a harmonization perspective, these patient-related determinants may therefore amplify residual differences between reagents and analytical systems even when procedural preanalytics are well controlled (*21*). They should therefore be considered biological modifiers of PT/INR interpretability rather than conventional preanalytical errors. This distinction is essential, because it explains why optimized collection and processing alone cannot ensure full PT/INR comparability across all clinical settings (*3*, *8*).

### Blood collection and phlebotomy-related variables

Proper venipuncture technique is fundamental to the reliability of PT/INR assays. Preanalytical errors occur in roughly 5% of coagulation specimen collections (*28*). Although the exact proportions reported in different studies differ, insufficient fill volume and clot formation consistently remain the predominant issues (*29*, *30*). In accordance with Clinical and Laboratory Standards Institute (CLSI) guidance, the recommended order of draw positions sodium citrate tubes (light blue, 3.2% citrate) immediately after blood culture bottles and before any serum, heparin, EDTA, or glycolytic inhibitor tubes (*31*). When a winged (“butterfly”) collection set is used, a discard tube (non-additive or citrate) must be drawn first to offset tubing dead space and avert under-filling; this discard remains mandatory even if only a coagulation tube is collected. No discard is required for standard evacuated tubes without a winged set.

Tourniquet application should not exceed one minute and must be released as soon as blood flow is established (*32*). Sodium citrate tubes must be filled to at least 90% of the nominal volume to maintain the critical 9:1 blood-to-anticoagulant ratio; under-filling leads to relative citrate excess and consequent falsely prolonged PT/INR values. Studies demonstrate that underfilling 3.8% sodium citrate collection tubes significantly prolongs PT (*33*). However, when using the 3.2% sodium citrate currently recommended by CLSI, no statistically significant difference in PT results was observed between 60% and 100% fill volumes (*31*). Immediately after collection, the tube should be gently inverted four to five times to ensure thorough mixing, whereas vigorous shaking should be avoided to prevent hemolysis (*34*). A clean, minimally traumatic venipuncture minimises tissue factor contamination, and severely hemolyzed samples are considered unacceptable (*35*). Collectively, strict adherence to these standardised procedures and to the correct order of draw effectively minimises collection-related variability (*36*).

### Anticoagulant ratio, processing, and sample stability

After collection, the *in vitro* specimen environment is critical. Prothrombin time depends on accurate reversal of citrate-mediated calcium chelation. In markedly elevated hematocrit (> 55%), reduced plasma fraction produces relative anticoagulant excess. Guidelines recommend adjusted citrate volumes, yet the evidence base derives predominantly from indirect studies of underfilled tubes or artificially constructed hematocrit values rather than direct patient data (*31*). A study demonstrated that the impact of citrate adjustment depends critically on reagent type: recombinant tissue factor thromboplastin showed strong agreement between adjusted and unadjusted INRs (R^2^ = 0.823; median 2.25 *vs* 2.22), with > 10% deviation only when hematocrit exceeded 62%, whereas rabbit brain thromboplastin exhibited only moderate correlation (R^2^ = 0.427) and a clinically significant median INR difference (2.51 *vs* 3.45), challenging the universal application of current guidelines (*37*). From a practical standpoint, manual CLSI H21 adjustment is burdensome in high-throughput laboratories, requiring individual patient hematocrit values, manual tube preparation, and careful labeling, each step introducing potential human error, while automation strategies such as pre-labeled adjusted tubes or barcode-integrated calculation systems remain underutilized in most routine settings. Laboratories thus face a trade-off between theoretical accuracy and operational reality.

Prothrombin time/INR stability is time- and temperature-dependent: CLSI recommends up to 24 hours at room temperature, though refrigerated storage data are nuanced (plasma PT/INR is stable for up to 24 hours at 4 °C in some studies; whole blood PT/INR can also be refrigerated for up to 24 hours *per* the updated guideline, which supersedes previous recommendations against refrigeration (*31*, *38*). For patients receiving unfractionated heparin, whole blood specimens must be centrifuged within 1 hour at room temperature, and plasma analysis completed within 4 hours of collection; delays beyond these thresholds may compromise reliability due to platelet factor 4 mediated heparin neutralization (*39*). Ultimately, adequate centrifugation, defined as centrifugation of capped citrated whole blood at 1500xg for no less than 15 minutes at room temperature (15-25 °C), preferably using a swing-out bucket rotor with the brake deactivated, to achieve platelet-poor plasma with a residual platelet count < 10,000/μL, and strict adherence to standardized collection, processing, and timing protocols remain essential prerequisites for inter-laboratory comparability (*40*, *41*). Major procedural sources of preanalytical variability affecting PT/INR are shown in Table 1.

**Table 1:** Major procedural sources of preanalytical variability affecting PT/INR

**Variation category**	**Source**	**Mechanism**	**Effect on PT/INR**	**Controllability**
Blood collection	Prolonged or difficult venipuncture (41)	Partial activation during sampling; specimen quality compromise	Unreliable PT/INR	High
Blood collection	Incorrect order of draw in open systems / line contamination (41)	Carry-over of anticoagulants or other additives	Prolonged or unreliable PT/INR, especially when contamination occurs	High
Sample processing	Underfilled citrate tube (41,42)	Excess citrate ® calcium over-chelation	Prolonged PT / elevated INR	High
Sample processing	Extreme hematocrit (> 55%) without citrate adjustment (42)	Relative excess of citrate in reduced plasma volume	Prolonged PT / elevated INR	Medium
Sample processing	Inadequate centrifugation (40,42)	Residual cellular components and platelet phospholipid	Potentially altered or less reliable PT/INR (direction assay-dependent)	High
Transport / storage	Delay in processing or inadequate temperature control (42)	Time- and condition-dependent alteration of coagulation factor/activity stability	Usually prolonged or less reliable PT/INR	High
PT/INR - prothrombin time/international normalized ratio. PT – prothrombin time.				

## Analytical phase

The analytical phase is a critical determinant of PT/INR comparability. Although the ISI framework was introduced to reduce variability attributable to differences in thromboplastin responsiveness, analytical harmonization remains incomplete in routine practice. Residual non-comparability may persist because differences in reagent composition, analyzer-specific clot detection principles, and context-dependent sample behavior are only partially corrected by INR transformation (*1*-*4*). Analytical disagreement should therefore be understood not simply as the result of isolated technical imperfections, but as the combined consequence of biological complexity and system-dependent measurement design. This issue is particularly important in multi-center clinical trials, transplant networks involving cross-border organ allocation, or regions with high population diversity. Laboratories should consider local MNPT establishment or formal transfer validation when patient demographics or analytical systems differ substantially from reference conditions (*41*).

### Thromboplastin reagents and analytical sensitivity

The limitations of the ISI model’s foundational assumptions become especially evident outside the classic vitamin VKA setting. The model presupposes coordinated declines in vitamin K-dependent factors, thereby allowing universal calibration correction. When factor abnormalities are non-parallel, the PT prolongation-to-deficiency relationship becomes reagent- and system-dependent. In advanced liver disease, disproportionate factor VII reduction produces reagent-dependent PT responses that cannot be fully corrected by a single ISI conversion (*17*, *42*).

Current guidelines recommend evaluating at least 20 normal plasmas and 60 stable VKA-treated samples spanning INR approximately 1.5-4.5, with log-transformed PT results compared to the reference system via orthogonal regression (*43*, *44*). To address method-dependent bias, Tripodi and colleagues developed INR calibrated for cirrhosis (INR(liver)), using a cirrhosis-specific ISI derived from chronic liver disease patients (*45*). Independent validation yielded mixed results: Bechmann *et al.* reported that INR(liver) reduced but did not eliminate method-related Model for End-Stage Liver Disease (MELD) variation, with residual 8-12% disagreement at INR > 2.0 (*46*). White *et al.* confirmed improvement was most pronounced at lower INR ranges (1.5-2.5) but diminished with severe factor VII depletion. These data indicate INR(liver) is a partial solution; clinical harmonization requires complementary strategies including ISI optimization and thrombin generation assay (TGA) integration (*47*, *48*). Similar divergence has been documented in sepsis-associated coagulopathy and other non-VKA conditions where factor consumption patterns are heterogeneous (*49*).

Another issue concerns MNPT sourcing. Laboratories may locally determine MNPT from healthy donors or, in some settings, rely on manufacturer assignments. The latter assumes manufacturer reference conditions match the local reagent-instrument combination, an assumption that may not hold in practice (*41*, *50*). Direct adoption of manufacturer-assigned MNPT may introduce systematic bias if local normal PT values differ. Postanalytical harmonization requires not only correct formula application but validation that the MNPT is suitable for the local measurement system.

### Detection principles and endpoint definition

Analytical variability also arises from the way clot formation is detected and translated into a reportable endpoint. Clotting time is assigned through platform-specific detection principles (Table 2). Prothrombin time systems use mechanical, viscosity-based, or optical methods. These differ in susceptibility to interference from hemolysis, lipemia, bilirubin, and hypofibrinogenemia (*13*).

**Table 2:** Detection principles in coagulation analyzers and their relevance to PT/INR

**Analytical aspect**	**Mechanical/viscosity-based detection**	**Optical detection**	**Selected magnetic bead-based platforms**	**Relevance to PT/INR comparability**
Physical principle	Detects changes in sample movement, oscillation, or viscosity as fibrin polymerization progresses	Detects changes in light transmission, absorbance, or turbidity during clot formation	Detects clot-related changes in magnetic particle motion using platform-specific sensing systems	Different detection principles may translate the same coagulation reaction into different PT seconds, which are not always fully normalized by INR conversion
Endpoint definition	Endpoint is assigned when movement attenuation or viscosity-related threshold criteria are reached	Endpoint is assigned from optical curve thresholds and platform-specific signal-processing algorithms	Endpoint is assigned from changes in magnetic particle behavior together with platform-specific threshold algorithms	Endpoint assignment is operational rather than universal, and may therefore contribute to inter-system variability
Susceptibility to hemolysis/lipemia/icterus	Less susceptible to purely optical interference, although biological and matrix-related effects may still influence results	More susceptible to optical interference, although modern HIL checks and correction algorithms may mitigate some effects	In some comparative studies, showed lower susceptibility to optical interference than photo-optical systems; performance remains platform-dependent	Matrix-dependent bias differs across systems and may reduce comparability in visually compromised samples
Performance in low fibrinogen states	Weak clot formation may still impair endpoint recognition, particularly in severe hypofibrinogenemia	Weak or shallow optical clotting curves may reduce signal robustness	Performance may remain acceptable on some platforms, but depends on assay design and signal-processing rules	Severe hypofibrinogenemia can accentuate system-dependent differences in clot endpoint assignment
Additional analytical output	Usually limited to endpoint-based clotting results	May provide full clotting curves and derived waveform parameters; clot waveform analysis is primarily associated with photo-optical systems	Usually limited in routine PT output, depending on platform implementation	Additional algorithmic output may improve interpretation, but also increases platform dependence
Practical implication	Often advantageous in optically compromised samples, but not inherently interchangeable with other systems	Informative and waveform-capable, but more sensitive to optical artifacts	Potentially useful in selected optically compromised specimens, but not inherently interchangeable with other systems	PT/INR harmonization should evaluate the reagent–instrument pair as the analytical unit rather than the reagent alone
PT/INR - prothrombin time/international normalized ratio. HIL - hemolysis/icterus/lipemia index. PT – prothrombin time.				

Traditional internal quality control (IQC) and EQA remain laboratory quality assurance foundations. However, satisfactory performance in traditional quality systems does not guarantee clinically equivalent results across laboratories, because PT/INR comparability depends not only on analytical precision but also on whether quality assessment materials behave like real patient plasma across different reagent-instrument systems (*51*).

Analytical non-comparability is therefore not reducible to reagent sensitivity alone. Instead, PT/INR performance should be understood at the level of the integrated analyzer–reagent system, in which the same biological reaction may be translated into different clotting times depending on how endpoint formation is detected and processed. For PT/INR harmonization, the relevant unit of comparison is thus not the reagent in isolation, but the full measurement system through which coagulation is measured and converted into reportable data (see Table 2).

### Pharmacodynamic interference from direct oral anticoagulants

The increasing use of DOACs illustrates an important boundary of PT/INR harmonization outside the VKA model. Unlike VKAs, which reduce several vitamin K-dependent clotting factors in a relatively coordinated manner, DOACs exert target-specific effects on coagulation assays (*14*). Their influence on PT therefore reflects drug-specific and reagent-dependent analytical effects rather than a biological pattern that can be reliably normalized through conventional INR transformation. A systematic review by Samuelson *et al.* demonstrated that the performance of standard anticoagulation tests varies across DOACs and reagents, with most assays showing insufficient correlation to provide a reliable assessment of DOAC effects (*16*).

However, recent developments include the MRX PT DOAC assay, which generates a DOAC Normalised Ratio (DNR) from a DOAC-sensitive PT and an INR from a DOAC-insensitive PT, with the Clot Time Ratio (CTR) calculated as DNR/INR (*52*). In a 2024 evaluation, the MRX PT DOAC assay demonstrated 100% sensitivity and 100% negative predictive value for edoxaban concentrations > 50 ng/mL, and can be run on widely available coagulation analyzers (*53*). This suggests that reagent-dependent DOAC effects may be harnessed for detection purposes rather than dismissed solely as confounding variability. Direct oral anticoagulant stop reagents, such as DOAC Stop and DP-Filter, offer another pathway: these products can effectively neutralize the *in vitro* effect of all DOACs, including rivaroxaban, enabling accurate baseline PT/INR determination in DOAC-exposed samples (*54*, *55*). Future guidelines may evolve to incorporate DOAC-specific verification protocols, particularly for emergency settings where rapid exclusion of clinically relevant DOAC concentrations is required. Typical effects of selected DOACs on PT assays and associated interpretation risks are shown in Table 3.

**Table 3:** Typical effects of selected DOACs on PT assays and associated interpretation risks

**Drug class/example**	**Typical effect on PT**	**Inter-reagent variability**	**Main interpretation risk**	**Recommended reporting implication**	**References**
Direct factor Xa inhibitor (rivaroxaban)	Often moderate to marked PT prolongation	High	PT prolongation may be misread as a standardized estimate of anticoagulant intensity	PT/INR should not be used to estimate rivaroxaban intensity; if assessment is clinically required, use a drug-appropriate assay	(*(**14**,**15**,**56**)*)
Direct factor Xa inhibitor (apixaban)	Usually minimal, absent, or inconsistent effect in many PT systems	High	A normal PT may falsely reassure clinicians despite clinically relevant drug exposure	A normal PT does not exclude clinically relevant apixaban exposure	(*(**14**,**15**,**57**)*)
Direct thrombin inhibitor (dabigatran)	Usually weak and inconsistent PT effect; prolongation is more likely at higher concentrations	Moderate to high	PT is relatively insensitive and may underestimate drug presence	PT is not suitable for excluding dabigatran; dilute thrombin time or ecarin-based assays are preferred when assessment is required	(*(**14**,**56**,**57**)*)
Direct factor Xa inhibitor (edoxaban)	Moderate PT prolongation, greater than apixaban but less than rivaroxaban in most systems (1.5-2.5 x control at peak)	Moderate to high	INR may appear falsely elevated; edoxaban-specific anti-Xa assay preferred for quantitative assessment	PT/INR should not be used to estimate edoxaban intensity; anti-Xa assay recommended if quantitative data required	(*(**14**,**56**)*)
DOACs overall	Drug-specific and reagent-dependent PT effects that do not conform to the VKA calibration model	High	INR reporting may give a misleading impression of standardization and comparability	PT results in DOAC-treated patients should not be interpreted within the conventional VKA-oriented INR framework	(*(**14**,**56**,**57**)*)
DOACs - direct oral anticoagulants. PT – prothrombin time. PT/INR - prothrombin time/international normalized ratio. VKA - vitamin K antagonist.					

### Artificial intelligence and machine learning in PT/INR quality assurance

Artificial intelligence (AI) and machine learning (ML) approaches have recently been explored in laboratory quality systems, particularly for patient-based real-time quality control (PBRTQC) and coagulation waveform analysis (*58*-*60*). Existing studies using ensemble learning and rule-based hybrid models suggest that ML may improve the early detection of analytical drift; however, the magnitude of improvement is strongly dependent on dataset structure, local patient mix, and calibration strategy, and cannot be generalized as a fixed performance gain across laboratories (*58*). For example, reported improvements in drift detection sensitivity vary widely across simulation-based and retrospective datasets, and are influenced by baseline analytical stability and signal-to-noise ratio rather than algorithm type alone (*58*, *59*). Consequently, performance estimates should be interpreted as context-specific rather than transferable metrics, and prospective multicenter validation remains limited.

Deep learning models applied to optical clot waveform data have demonstrated promising capability in identifying preanalytical interference such as hemolysis, lipemia, and icterus. Nevertheless, most published studies remain retrospective or single-platform in design, and clinical implementation requires validation across heterogeneous analyzer-reagent systems before routine adoption (*61*). Importantly, current evidence supports AI as an adjunctive interpretive layer rather than a replacement for standardized analytical quality systems. Integration into routine laboratory workflows will require resolution of key barriers, including model transparency, regulatory validation, interoperability with laboratory information systems, and dataset harmonization across institutions (*60*, *62*).

### Point-of-care testing INR devices and clinical harmonization implication

The expansion of point-of-care testing (POCT) has extended the PT/INR harmonization challenge beyond centralized laboratories into decentralized clinical settings (*63*). While POCT INR devices improve care accessibility and enable rapid feedback for outpatient and home VKA monitoring, they cannot be assumed analytically interchangeable with central laboratory assays without formal verification (*64*). Point-of-care testing systems adopt strip- or cartridge-based whole-blood detection, whereas laboratory assays use platelet-poor plasma with optical or mechanical clot detection, creating inherent gaps in detection principle and matrix susceptibility (*65*, *66*).

Agreement between the two methods is generally acceptable within conventional therapeutic INR ranges, but bias progressively increases at higher values, driven by analytical non-linearity and intrinsic matrix effects of whole-blood testing (*67*). Discordance risk is further amplified in patients with advanced liver disease or abnormal hematocrit and fibrinogen concentrations. This carries clinical relevance: MELD scores are standardized to laboratory INR, while POCT results commonly seen in emergency or ambulatory settings may lead to misclassification of disease severity (*63*, *68*).

Regular comparative verification against local central laboratory systems is essential. Current guidance recommends method comparison in stable VKA-treated patients, with an acceptable paired deviation of approximately 0.5 INR units (*69*). Venous laboratory confirmation should be prioritized before major clinical decisions at high INR levels (*65*). U.S. Food and Drug Administration (FDA) recalls of INRatio devices further underscore the necessity of device-specific validation, even as newer platforms show improved performance (*67*, *70*). Point-of-care testing does not undermine the broader INR framework, but clarifies that cross-system comparability remains conditional on measurement method, specimen matrix, and clinical context.

### Owren-Quick Divergence and Fiix-PT Calibration

The persistent methodological divergence between Owren and Quick PT systems remains a relevant source of inter-laboratory variability despite shared ISI-based calibration (*71*). Although INR transformation improves comparability, residual bias has been consistently demonstrated at higher INR ranges and in complex patient populations, particularly in the context of liver disease and intensive anticoagulation regimens (*68*).

The Fiix-PT concept offers an innovative approach to reducing analytical variability by selectively prioritizing factor II and X activity while attenuating sensitivity to factor VII-driven fluctuations (*72*). This strategy may enhance analytical stability in VKA monitoring; however, its clinical adoption remains at an early validation stage, with current evidence largely derived from observational and regionally confined studies (*73*). Notably, while the Icelandic nationwide cohort study demonstrated improved VKA stability and reduced bleeding events, these findings were obtained within a relatively homogeneous healthcare system with centralized laboratory standardization, which may limit their direct extrapolation to more heterogeneous international settings (*74*).

Accordingly, Fiix-PT should currently be regarded as a promising yet context-sensitive calibration strategy, requiring robust multicenter validation across diverse reagent–instrument systems before broad implementation can be recommended (*73*). From a harmonization perspective, it is more appropriately viewed as part of a broader conceptual evolution toward mechanism-informed coagulation assessment rather than as a direct substitute for conventional PT/INR. Future research should prioritize: (i) multicenter validation across different reagent classes; (ii) head-to-head comparisons with standardized INR in diverse populations; and (iii) integration into clinical decision algorithms, rather than relying on isolated numerical substitution.

## Postanalytical phase

Postanalytical variability in PT/INR testing arises mainly from the parameters used to convert PT seconds into INR, the compatibility of those parameters with the local reagent–instrument system, and the way INR results are subsequently interpreted or incorporated into clinical decision tools (*43*). Although these factors occur after clot detection, they can introduce clinically relevant bias because INR is a calculated result rather than a directly measured analyte.

### Determination of the mean normal prothrombin time

Mean normal prothrombin time serves as the baseline denominator in the INR formula, and systematic bias in this value propagates directly into all patient INR results (*43*). Manufacturers typically provide a default MNPT value established under reference conditions, but this value may not transfer reliably to local reagent-instrument combinations or patient populations. Population-related variation in normal PT values represents an often-overlooked source of systematic bias: reference intervals for coagulation tests established in Western cohorts may not be appropriate for non-Western populations, and direct adoption of imported MNPT values can introduce consistent offset across all patient results (*75*).

Current CLSI guidelines recommend that laboratories establish or verify MNPT locally using at least 20 plasma samples from healthy donors matched to the local patient demographic (*43*). For open analytical systems where reagents are used on non-validated instrument platforms, local MNPT determination is not optional but a methodological requirement to maintain metrological traceability. Field evidence indicates that laboratories performing local MNPT verification exhibit significantly lower between-laboratory INR bias than those relying exclusively on manufacturer-assigned values (*76*). In multinational clinical trials or cross-border organ allocation networks, formal transfer validation of MNPT is particularly critical, as even small deviations in baseline PT can produce clinically meaningful INR differences near therapeutic or prognostic thresholds.

### International Sensitivity Index verification and postanalytical system compatibility

The ISI is the core calibration parameter that normalizes thromboplastin sensitivity differences across reagents. However, manufacturer-assigned ISI values are validated only for specific reagent-instrument combinations; when reagents are deployed on alternative platforms in open systems, the ISI may no longer be accurate, introducing systematic bias into INR calculations (*3*, *77*).

*Per* CLSI H54-A guidelines, local ISI calibration or verification requires testing at least 20 normal plasmas and 60 stable VKA-treated samples spanning an INR range of approximately 1.5-4.5, with orthogonal regression applied to log-transformed PT results against a reference measurement system (*43*). This procedure corrects for platform-specific differences in clot endpoint detection, optical signal processing, and reagent-instrument interaction that cannot be captured by a generic reagent ISI. Multi-center evaluation data demonstrate that local ISI calibration reduces between-laboratory INR bias compared with using manufacturer-assigned values alone, particularly at higher INR values (*78*). Despite this well-documented benefit, local calibration remains underimplemented in routine practice, contributing to persistent postanalytical variability across institutions.

Postanalytical harmonization therefore requires recognition that INR is generated by an integrated reagent-instrument-calculation system, not by reagent sensitivity alone. Regulatory frameworks increasingly emphasize that manufacturers should provide system-specific ISI values for each certified reagent-instrument pair, rather than a single generic reagent ISI, to reduce the burden on individual laboratories and improve baseline standardization.

### Clinical amplification of INR variability

The clinical impact of postanalytical INR variability is disproportionately magnified when INR values are incorporated into validated prognostic scoring systems, most notably the MELD score (*68*). The MELD algorithm uses a log-transformed INR term, meaning that even small absolute differences in INR (0.2-0.3 units) can alter calculated disease severity and shift patient priority near liver transplantation eligibility thresholds (*79*).

This amplification effect is particularly consequential in advanced liver disease, where INR itself is already subject to substantial reagent-dependent analytical bias due to non-parallel factor deficiency (*25*). Postanalytical errors in MNPT or ISI thus compound pre-existing analytical non-equivalence, leading to clinically significant misclassification of cirrhosis severity. To minimize this cumulative variation, laboratories serving liver transplant centers are advised to prioritize thromboplastin reagents with ISI values closest to 1.0, implement local ISI/MNPT calibration, and participate in EQA programs using commutable human plasma materials (*25*, *80*).

## Future outlooks

Although the INR system has substantially improved comparability of PT results for monitoring vitamin K antagonist therapy, complete harmonization across laboratories remains difficult (*5*, *8*). Future research should focus on four areas: local verification of MNPT and ISI in open reagent-instrument systems; broader use of commutable EQA materials; indication-specific interpretation for clinical settings in which conventional INR assumptions are not fulfilled; and prospective validation of complementary approaches such as Fiix-PT, thrombin generation assays, PBRTQC, and AI-assisted quality surveillance. These priorities require multicentre studies including diverse patient populations, different thromboplastin reagents, Quick and Owren methods, central-laboratory and decentralized testing environments, and clinically relevant outcomes.

## Conclusion

The INR remains one of the most influential standardization tools in laboratory medicine, but its success should not obscure its limitations. As this review shows, calibration of thromboplastin responsiveness alone does not ensure full PT comparability across the total testing process or across all clinical settings. Residual variability may arise from patient-related biological context, specimen handling, reagent-instrument interactions, postanalytical parameter validity, and decentralized testing conditions. Prothrombin time/INR harmonization should therefore be understood not as a completed achievement, but as an ongoing process requiring integrated control across preanalytical, analytical, and postanalytical phases together with quality systems that reflect real-world patient samples and routine practice.

## Data Availability

No data was generated during this study, so data sharing statement is not applicable to this article.
